# Chronic intermittent ethanol exposure during adolescence produces sex- and age-dependent changes in anxiety and cognition without changes in microglia reactivity late in life

**DOI:** 10.3389/fnbeh.2023.1223883

**Published:** 2023-08-01

**Authors:** Douglas B. Matthews, Samantha Scaletty, Sarah Trapp, Areonna Schreiber, Gillian Rossmann, Bailey Imhoff, Quinn Petersilka, Abigail Kastner, Jim Pauly, Kimberly Nixon

**Affiliations:** ^1^Department of Psychology, University of Wisconsin–Eau Claire, Eau Claire, WI, United States; ^2^Department of Pharmaceutical Sciences, College of Pharmacy, University of Kentucky, Lexington, KY, United States; ^3^Division of Pharmacology and Toxicology, College of Pharmacy, The University of Texas at Austin, Austin, TX, United States

**Keywords:** adolescence, aging, behavioral flexibility, chronic intermittent ethanol, microglia

## Abstract

Binge-like ethanol exposure during adolescence has been shown to produce long lasting effects in animal models including anxiety-like behavior that can last into young adulthood and impairments in cognition that can last throughout most of the lifespan. However, little research has investigated if binge-like ethanol exposure during adolescence produces persistent anxiety-like behavior and concomitantly impairs cognition late in life. Furthermore, few studies have investigated such behavioral effects in both female and male rats over the lifespan. Finally, it is yet to be determined if binge-like ethanol exposure during adolescence alters microglia activation in relevant brain regions late in life. In the present study female and male adolescent rats were exposed to either 3.0 or 5.0 g/kg ethanol, or water control, in a chronic intermittent pattern before being tested in the elevated plus maze and open field task over the next ∼18 months. Animals were then trained in a spatial reference task via the Morris water maze before having their behavioral flexibility tested. Finally, brains were removed, sectioned and presumptive microglia activation determined using autoradiography for [^3^H]PK11195 binding. Males, but not females, displayed an anxiety-like phenotype initially following the chronic intermittent ethanol exposure paradigm which resolved in adulthood. Further, males but not females had altered spatial reference learning and impaired behavioral flexibility late in life. Conversely, [^3^H]PK11195 binding was significantly elevated in females compared to males late in life and the level of microglia activation interacted as a function of sex and brain regions, but there was no long-term outcome related to adolescent alcohol exposure. These data further confirm that binge-like ethanol exposure during adolescence produces alterations in behavior that can last throughout the lifespan. In addition, the data suggest that microglia activation late in life is not exacerbated by prior binge-like ethanol exposure during adolescence but the expression is sex- and brain region-dependent across the lifespan.

## Introduction

Alcohol (ethanol) is one of the most used and misused drugs in the world (Ferreira and Willoughby, 2008) resulting in great personal and financial loss (Manthey et al., 2021). Recent data suggest that alcohol use is increasing due to the COVID-19 pandemic in most age groups including adolescents (Pollard et al., 2020) and the increased alcohol use in general is resulting in greater alcohol-related deaths (White et al., 2022). While it is often reported that adolescents consume alcohol at high levels and in a dangerous, binge fashion (Patrick and Terry-McElrath, 2019; U.S. Department of Health and Human Services, 2019), current world health concerns have likely exacerbated adolescent alcohol use and misuse (Dodge et al., 2021). Consequently, understanding the impact of binge alcohol use during adolescence across the lifespan is a critical public health need.

Alcohol consumption during adolescence can have life-long consequences. For example, chronic intermittent ethanol (CIE) during adolescence will block the age-related increase in the hypnotic effects of high dose ethanol (Silvers et al., 2003; Matthews et al., 2008) and reduce ethanol-induced hypothermia (Swartzwelder et al., 1998; White et al., 2002). Furthermore, CIE during adolescence can produce effects that last into young adulthood. For example, CIE during adolescence can produce anxiety-like behaviors that last into adulthood (Coleman et al., 2014; Van Skike et al., 2015; Varlinskaya et al., 2015; Vetreno et al., 2016; Kokare et al., 2017; Lee et al., 2017). However, the vast majority of these studies have only used male subjects and there are a limited number of studies that have included both female and male subjects (see Robinson et al., 2021 for a review). For example, male rats, but not female rats, exposed to chronic ethanol early in adolescence have a social anxiety-like phenotype (Varlinskaya et al., 2015). Furthermore, both male and female subjects exposed to ethanol early in adolescence have increases in anxiety-like behavior in the elevated plus maze, while only males have an anxiety-like phenotype following ethanol exposure later in adolescence (Towner and Varlinskaya, 2020). Finally, a recent study has shown that chronic exposure to ethanol during adolescence can produce a sex-specific effect on the elevated plus maze, but this effect is driven by an increased amount of time on the open arms in females (Healey et al., 2021). Conversely, other studies have found minimal effects of chronic ethanol exposure during adolescence on anxiety-like behaviors (Amodeo et al., 2018; Gamble and Diaz, 2020). Given the non-uniform results on the sex-specific effects of CIE on anxiety-like behavior coupled with the lack of studies investigating the impact of CIE on anxiety-like behaviors late in life, additional research in this area is needed.

Chronic intermittent ethanol exposure (CIEg) during adolescence may also impair cognition (Reitz et al., 2021), specifically behavioral flexibility, in young adulthood (Coleman et al., 2014; Gass et al., 2014; Barker et al., 2017; Fernandez et al., 2017; Contreras et al., 2019; Sey et al., 2019; Macht et al., 2020). Behavioral flexibility, the ability to learn something new in the face of old learning contingencies, has been identified as a critical cognitive measure that correlates with ethanol consumption in abstinent alcoholics and predicts ethanol consumption in non-human primate models (Shnitko et al., 2020). However, while several studies have investigated if CIE during adolescence impairs cognition and behavioral flexibility in young adulthood, only a single study investigated if CIE during adolescence impairs behavioral flexibility beyond young adulthood into older life stages (Matthews et al., 2022). In addition, few studies have investigated sex differences in cognition and behavioral flexibility in late adulthood or in aging following CIE during adolescence.

The average age of the world’s population is increasing, and it has been predicted by the year 2050, the number of adults over the age of 65 will double. Older adults continue to consume alcohol often in a dangerous binge pattern (Keyes, 2023) and it has been suggested that understanding the impact of alcohol use in the older population is a critical public health concern (Matthews and Koob, 2023; Matthews and Rossmann, 2023; White et al., 2023). Unfortunately, while much research has investigated the lasting behavioral effects of CIE during adolescence into young adulthood (see Crews et al., 2019 for review), little is known how CIE during adolescence impacts aged animals.

Two previous studies from our laboratory subjected male rats or male and female rats to CIE during adolescence and then tested them on a variety of behaviors several times over the next 18–22 months (Matthews et al., 2017, 2022). In the first study (Matthews et al., 2017), CIE administration during adolescence produced both long-lasting (over 500 days after CIE during adolescence) tolerance to a high dose ethanol challenge, as measured by sleep time, while also producing sensitization to ethanol-induced spatial memory impairments, an impairment likely driven by altered hippocampal function (see Van Skike et al., 2019 for a review of this literature). In the second study (Matthews et al., 2022), CIE during adolescence impaired behavioral flexibility in male and female rats but the pattern of behavior was different between the two sexes. Specifically, CIE during adolescence interacted with age in male rats to impair behavioral flexibility primarily later in life and only in subjects administered 5.0 g/kg ethanol during the adolescent exposure period while CIE during adolescence impaired behavioral flexibility across the lifespan in female subjects. Given the brain and hippocampus are still developing during adolescence (Bayer et al., 1982), these data suggest that exposure to ethanol in a chronic binge-like fashion during adolescence may produce changes which alter behavior across the entirety of the lifespan. However, these studies left several important issues unanswered. First, there was limited investigation into how CIE during adolescence impacted behavior in females later in life. Second, the studies did not investigate if CIE during adolescence produced a long-lasting anxiety-like effect in either male and/or female rats. Third, it is unknown if cognitive performance to a novel test late in life is differentially impacted by sex of subject and CIE during adolescence. Finally, no corresponding neurobiological measure was assessed to gain information into possible mechanisms mediating the reported behavioral changes.

Microglia are innate glial cells that play a variety of roles in the central nervous system including in neurodevelopment, synaptic pruning and plasticity (see Salter and Stevens, 2017 for review). Microglia also react to insults such as those achieved with binge-like ethanol administration (see Melbourne et al., 2021 for review). For example, postmortem analysis of brains from humans with an AUD have shown reactive microglia (He and Crews, 2008) and this effect may be age dependent (Carlson et al., 2023). We have shown that alcohol exposure during adolescence causes a microglia reaction (McClain et al., 2011; Peng and Nixon, 2021) that persists at least a month if not several months according to our adult models (Obernier et al., 2002; Nixon et al., 2008; see also Melbourne et al., 2021 for review). Therefore, it is possible that exposure to ethanol during adolescence may persistently alter microglia phenotype and state later in life.

The current project is designed to investigate if CIE during adolescence in both female and male rats produce alterations in anxiety-like behavior across the lifespan and impairs cognition, including behavioral flexibility, 19 months following CIE exposure during adolescence. Finally, as microglia effects can be long lasting, we used autoradiography for [^3^H]PK11195 binding to the translocator protein 18 kDa (TSPO) to measure neuroimmune activation and microglial reactivity (Chen and Guilarte, 2008). The TSPO is expressed on the outer membrane of mitochondria and therefore contributes to various mitochondrial functions such as respiration, opening of the mitochondrial permeability transition pore and putatively, cholesterol transport, for which it is named (Chen and Guilarte, 2008; Rupprecht et al., 2022). Autoradiography for TSPO has long been used to identify neuroimmune reactivity and especially microglia reactivity following insult (Chen and Guilarte, 2008). [^3^H]PK11195 binding has been a highly sensitive indicator of microglia effects in alcohol models (Marshall et al., 2013; Tyler et al., 2019) and one that persists at least months after alcohol exposure (Obernier et al., 2002). Therefore, we chose this approach to increase our likelihood of detecting long-term effects in brain tissue as microglia reactivity increases with aging, neurodegeneration, and excessive alcohol use (Carlson et al., 2023). Accordingly, [^3^H]PK11195 was examined in three brain regions important for spatial learning (entorhinal cortex, dentate gyrus, and CA1 region of the hippocampus) from female and male ethanol treated animals.

## Materials and methods

### Subjects

Forty-two male (mean body weight at the start of treatment was 78.06 g) and 42 female (mean body weight at the start of the treatment was 63.2 g) Sprague-Dawley rats (Envigo, Indianapolis, IN, USA) were used to investigate the effect of CIEg via gavage during adolescence on anxiety-like behavior across the lifespan and cognition and microglia activation late in life. Female subjects arrived in the colony on PND 28 and males arrived on week ending PND 28 and 2 days later animals from both sexes were pseudo-randomly divided into one of two ethanol conditions or a control condition (see below) for treatment during adolescence (e.g., PND 30–PND 48). Animal care procedures followed the guidelines of the University of Wisconsin–Eau Claire IACUC. Food and water were provided *ad libitum* except for the water control groups during the CIEg treatment period where food access was controlled to yoke the body weight of the control groups to the body weight of the high dose ethanol group to minimize any difference (see below). Animals were housed two subjects per cage in an Ecoflo system (Allentown Caging, Allentown, NJ, USA) on a 12:12 light:dark cycle (lights on at 6:00 am; lights off at 6:00 pm). All animal procedures occurred between 8:00 am and 2 pm on test days.

### Chronic intermittent ethanol exposure

As previously described (Matthews et al., 2022), 2 days after arriving in the colony, animals were randomly divided into one of two ethanol groups or one control group and received either ethanol [3.0 g/kg (*n* = 14 for male and female) or 5.0 g/kg (*n* = 14 for male and female)] or water [water amount was matched in volume to the 5.0 g/kg ethanol amount (*n* = 14 for male and female)]. Ethanol, 35% v/v, was administered via gavage every 48 h for 20 days, for a total of 10 intoxications and withdrawals. During treatment, all animals in the CIEg exposure groups had unrestricted access to food and water; all animals that received water gavage (i.e., the control group) were weight yoked to the average 5.0 g/kg CIEg-treated rats’ weight to control for ethanol-induced weight suppression. Animals were weighed daily as an indirect index of general health while CIEg was ongoing and then every 1–2 weeks throughout the study.

### Blood ethanol concentration

As previously reported in Matthews et al. (2022; a time when the current project was under investigation using the identical CIEg procedure), six male and three female animals underwent similar CIEg treatment at each of the ethanol doses to serve as BEC sentinels. For these subjects, the tail was nicked 60 min following gavage on the last treatment day and approximately 5 μl of blood was collected, centrifuged to separate the plasma, and blood ethanol concentrations were determined via an AM-1 Analox machine (North Yorkshire, United Kingdom) following manufacturing guidelines. As reported in Matthews et al. (2022), blood ethanol levels were within previously reported ranges and not significantly different by sex [males: 134 mg/dl (3.0 g/kg) and 165 mg/dl (5.0 g/kg); females: 134 mg/dl (3.0 g/kg) and 149 m/dl (5.0 g/kg)].

### Impact of CIEg during adolescence on anxiety and cognition

All subjects underwent a measure of anxiety-like behavior via the elevated plus maze on PND 49 before an additional measure of anxiety-like behavior in the open field at PND 224. Subjects were then allowed to age until PND 579 when anxiety-like behavior was reassessed via the elevated plus maze. Three days following the last elevated plus maze test (PND 582) animals underwent a spatial learning paradigm for 14 days followed by a 2-day behavioral flexibility test via a reversal paradigm.

#### Elevated plus maze

Twenty-four hours after the final ethanol-exposure (PND 49) and approximately 18 months later (PND 579), anxiety-like behaviors were measured on an elevated plus maze (Any-maze, Stoelting Co., Wood Dale, IL, USA) located in a behavioral room isolated from animal caging and housing as described previously (Matthews et al., 2019). Briefly, the apparatus was elevated 50 cm from the ground and consisted of four arms 50 cm in length and 10 cm wide. The walls of the closed arms were 40 cm in height and located at opposing sides of the maze. Light levels were approximately 12 LUX in the closed arms and 74 LUX in the open arms. Animals were moved to the testing room 10–20 min prior to experimentation for acclimation. Each animal was placed in the central location facing an open arm and allowed 5 min to explore the maze. Each animal was videotaped and data was analyzed later by two research teams of two experimenters blind to the CIEg condition. The apparatus was wiped clean with a dilute ethanol solution (10%) between trials. The data was averaged across scores of the two teams for open arm entries, closed arm entries, total movement (open arm entries + closed arm entries + middle area entries), percent open arm entries (open arm entries divided by open arm entries + closed arm entries × 100) and percent open arm time [open arm time (in seconds) divided by 300 × 100]. In order to be defined as an entry, all four paws had to be within a maze compartment. If an animal fell off an arm, it was replaced on the arm and run for the remaining time, but its data was not included in analysis as recommended (Walf and Frye, 2007).

#### Open field

On PND 224, subjects were assessed for anxiety-like behavior in the open field (Any-maze, Stoelting Co., Wood Dale, IL, USA) located in a behavioral room isolated from animal caging and housing and different than the room used for the elevated plus maze. Light levels were similar to the levels for the open arms of the elevated plus maze. The open field was a 39” per side square and had 12” tall clear Plexiglas sides. Animals were placed in the center of the open field and total movement and thigmotaxis was monitored via a video camera and data collected with ANY-maze video tracking system, version 5.3 (Stoelting Co., Wood Dale, IL, USA). Animals were allowed to explore the open field for 5 min before being removed and place in their home cage. The field was cleaned between subjects with a dilute (10%) ethanol solution.

#### Spatial learning and behavioral flexibility

On PND 582, the impact of CIEg during adolescence on reference spatial memory and behavioral flexibility was tested in the Morris water maze (Morris, 1981). Animals were trained in the standard spatial, submerged platform, task in the same room used for the open field test. Spatial learning occurred for 14 days, four trials per day, with each start originating from one of the compass locations and the order of the start locations was counterbalanced over days. The water tank had a diameter of 6-ft and was painted white while the water was made opaque by the addition of white, non-toxic Tempura watercolor paint. The escape platform was made of clear Plexiglas and was submerged ∼1-inch below the water surface. Each trial had a maximum time of 45 s. If the animal did not find the platform within that time, the subject was gently guided to the platform and allowed to stay on the platform for ∼5 s. Learning was assessed via a video camera and swim latency and swim pathlength was collected via the ANY-maze system, version 5.3. Following the 14-day spatial reference learning task, the platform was rotated 180° and animals received 2 days of additional training to reflect behavioral flexibility.

#### Tissue harvesting and [^3^H]PK11195 autoradiography

We chose [^3^H]PK11195 autoradiography for the TSPO for its sensitivity to microglial reactions in rat models of an alcohol use disorder (Marshall et al., 2013; Tyler et al., 2019). While not fully specific to microglia [see extensive discussion of this point (Guilarte et al., 2022)], increases in [^3^H]PK11195 binding via autoradiography parallel our microglia reactions and not our astrocyte reactions (Marshall et al., 2013; Peng and Nixon, 2021). Thus, 1 day following completion of the behavioral flexibility test, animals were rapidly decapitated and the whole brain removed and snap frozen in isopentane maintained at ∼−30°C. Brains (*n* = 5 per sex per dose) were shipped on dry ice to the University of Kentucky (J. R. Pauly Laboratory for processing). Brains were coded and consequently experimenters were blind to treatment groups throughout processing and analysis. Brains were cut on a cryostat (Leica CM1950) at 16 μm with and stored in a −80°C freezer. TSPO autoradiography was performed as previously reported for rat TBI and 4-day binge alcohol exposure (Kelso et al., 2006, 2009; Guseva et al., 2008; Marshall et al., 2013). The slides were removed from −80°C and allowed to thaw overnight and loaded into binding racks. The slides were incubated in the following buffers at 4°C: 50 mM Tris–HCL (pH 7.4) for 15 min, 50 mM Tris–HCL and 2 nM [^3^H]PK11195 (PerkinElmer, Boston, MA, USA, specific activity = 73.6 Ci/mmol) for 2 h, 3 washes in 50 mM Tris–HCL (pH 7.4) for 3 min each, and a brief wash in dd H2O. The slides were left overnight to dry at room temperature, placed into autoradiography cassettes and exposed to Kodak BioMax film for approximately 12 weeks. Digital images were captured and analyzed using ImageJ. Brain regions were analyzed bilaterally at Bregma −3.8 mm (Paxinos and Watson, 2013) and the uncalibrated optical densities were averaged.

### Statistical analysis

Our statistical strategy was focused on investigating if CIEg via gavage during adolescence altered anxiety-like behavior across the lifespan and cognitive performance and microglia activation late in life. As such, we are primarily focused on the interaction between ethanol exposure and measures of anxiety-like behavior and cognition. Given previous research has shown that there are age and sex dependent effects in rats when tested on the elevated plus maze (Johnston and File, 1991; Imhof et al., 1993) and learning spatial tasks in the Morris water maze (Beiko et al., 2004; Vorhees and Williams, 2014) we first verified if significant differences existed in our first elevated plus maze experiment (see below) then analyzed females and males separately due the underlying behavioral differences. Consequently, to address our research interest, we utilized Kruskal–Wallis tests for anxiety measurements with appropriate *post hoc* analysis (Dunn’s multiple test) for each of the elevated plus maze and open field tests. For cognitive tests, we used ANOVA with appropriate *post-hoc* tests to allow for repeated measures analysis due to the training procedure. For autoradiography of microglia reactivity, aged female and male rats were analyzed first to determine if sex-dependent differences existed in aged animals across the brain regions measured and then second to investigate if CIEg during adolescence altered the density of [^3^H]PK11195 for each sex independently. For all behavioral analysis, only animals that survived the ∼19-month experiment were included. Furthermore, given that experiments were conducted over 19 months and some animals died at various time points within that period, the number of subjects by sex by ethanol condition for each experiment is given with each data set result.

## Results

### Impact of chronic intermittent ethanol exposure on survival

Animals were removed from the study for one of two reasons. First, subjects died by natural causes. Second, animals were removed from the study due to sudden, significant health issues which generally included either sudden weight loss or the development of a growth/tumor that was large enough to pose a health risk to the subject.

Previously (Matthews et al., 2022) we reported differential survivability between males and females administered ethanol via CIEg during adolescence over a 22-month test period. To investigate the impact of chronic ethanol on survivability, we first sought to ensure survivability did not differ in the water treated animals via log-rank Mantel-Cox tests. Specifically, we calculated survival curves for male and female subjects that were treated with water during the adolescent treatment period to investigate if sex produced differential survival during the experiment. As expected, no significant difference in survival was found between females and males that were administered water during adolescence (log-rank Mantel-Cox tests, *p* > 0.10). We next investigated if survival differences existed in males administered either 3.0 or 5.0 g/kg ethanol and no significant difference was found (log-rank Mantel-Cox tests, *p* > 0.10). We then investigated if survival differences existed in females administered either 3.0 or 5.0 g/kg ethanol and found no significant difference on survival (log-rank Mantel-Cox tests, *p* > 0.10). Given the lack of effect on survival in both males administered ethanol and females administered ethanol, we combined the males exposed to either 3.0 or 5.0 g/kg ethanol into one group and the females exposed to 3.0 or 5.0 g/kg ethanol into another group and queried survival curves between the two sexes. Once again, no significant difference in survivability was found between males and females administered CIE during adolescence during the ∼19-month experiment (log-rank Mantel-Cox tests, *p* > 0.10) (data not shown).

### Impact of chronic intermittent ethanol exposure on bodyweight

#### Males

The average body weight of animals did not differ prior to ethanol, or water, treatment (one-way ANOVA, *p* > 0.05). Similar to previous results (Matthews and Mittleman, 2017), during the CIEg treatment, body weight was significantly reduced by ethanol exposure [two-way ANOVA with repeated measures, CIEg dose (3) by treatment day (10), significant interaction of CIEg dose by treatment day, *F* = 6.2, df(18,270), *p* < 0.0001]. *Post hoc* analysis with the bodyweight of the water gavage group set as the control group, revealed that food yoking between the control group and the animals administered 5.0 g/kg ethanol was successful except for the third ethanol exposure day where the control treated animals weighed less than the 5.0 g/kg ethanol treated animals (Dunnett’s test, *p* = 0.0076). Furthermore, animals administered 3.0 g/kg ethanol weighed significantly more than control animals on CIEg treatment day 7 (Dunnett’s, *p* < 0.0014). Further, to assess the impact of CIEg during adolescence on animals’ bodyweights, subjects were weighed either weekly or every other week (during COVID-19 restrictions). Unlike the bodyweights during the treatment, CIEg during adolescence did not significantly alter the bodyweight of animals in either the 3.0 or 5.0 g/kg condition compared to the control condition [two-way ANOVA with repeated measures, main effect of day, *F* = 778.5, df(136,2040), *p* < 0.0001] (data not shown).

#### Females

The average weight of animals in the different ethanol conditions (or control) did not differ at the start of the experiment (one-way ANOVA, *p* > 0.05). Similar to what was found with males during the CIEg treatment, it was found that body weight was significantly impacted by ethanol exposure (two-way ANOVA with repeated measures, ethanol dose (3) by treatment day (10), significant interaction of ethanol dose by day, *F* = 2.068, df(18,320), *p* = 0.0069]. *Post hoc* analysis with the bodyweight of the water gavage group set as the control group, revealed that food yoking between the control group and the animals administered 5.0 g/kg ethanol was successful except for treatment day 2 (Dunnett’s test, *p* = 0.03), day 3 (Dunnett’s test, *p* = 0.009), and day 5 (Dunnett’s test, *p* = 0.0026) where the control treated animals weighed less than the 5.0 g/kg animals. In addition, animals administered 3.0 g/kg ethanol weighed significantly more than control animals on CIEg treatment day 2 (Dunnett’s test, *p* = 0.0347) and day 4 (Dunnett’s test, *p* = 0.0068). Finally, and similar to what was found with males, CIEg during adolescence did not alter bodyweights after completion of the treatment and throughout the lifespan [two-way ANOVA, ethanol dose by day, main effect of day, *F* = 135.5, df(64,2048), *p* < 0.0001] (data not shown).

### Impact of chronic intermittent ethanol exposure during adolescence on anxiety-like behavior

We first verified that a sex difference in anxiety-like behavior existed in the first plus maze test as expected from previous literature (Johnston and File, 1991; Imhof et al., 1993) by analyzing males and females exposed to water (the control subjects). A sex difference was found in that males had greater anxiety-like behavior on the elevated plus maze as evidenced by significantly less percent open arm time compared to females (Mann–Whitney *U* test, *U* = 25, *p* = 0.0053). We therefore separated analysis by sex of subject.

#### Males

**FIGURE 1:**
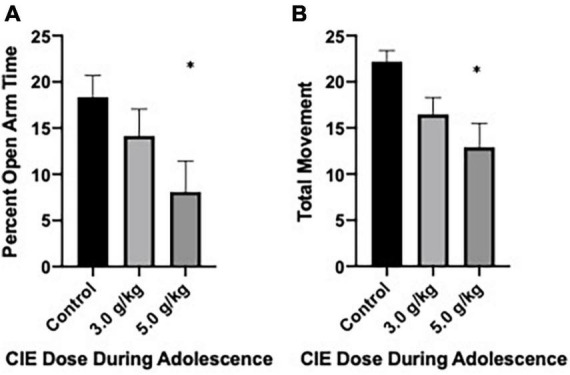
Chronic intermittent ethanol exposure via gavage during adolescence produces anxiety-like behavior in male rats when tested at PND 49. Male rats tested on the elevated plus maze on PND 49 demonstrated an anxiety-like effect as measured by percent open arm time **(A)** or total movement **(B)**. Data are mean with error bars denoting standard error of the mean. The symbol “*” denote *p* < 0.05, see text for exact value.

Anxiety-like behavior following CIEg exposure during adolescence was first assessed via the elevated plus maze on PND 49. After accounting for subjects that either A. were not alive at this time point or B. fell off the maze, a total of 35 subjects (control, *n* = 12; 3.0 g/kg ethanol *n* = 13; 5.0 g/kg ethanol *n* = 10) were run in the experiment. CIEg during adolescence produced a significant anxiogenic-like phenotype as measured by percent open arm time in male rats (Kruskal–Wallis test, KW = 6.48, *p* = 0.0392). *Post hoc* analysis revealed that animals administered 5.0 g/kg ethanol during adolescence spent significantly less percent time on the open arms compared to control animals (Dunn’s multiple comparisons test, *z* = 2.546, *p* = 0.0218). However, no significant effect for percent open arm entries was found, although a trend existed where subjects administered 5.0 g/kg ethanol during adolescence had fewer open arm entries compared to control animals. Finally, CIEg during adolescence significantly reduced total movement (Kruskal–Wallis test, KW = 8.809, *p* = 0.0122). Once again, *post-hoc* tests revealed that animals administered 5.0 g/kg ethanol during adolescence had less total movement compared to control animals (Dunn’s multiple comparisons test, *p* = 0.009) (see Figure 1).

Open field was determined on PND 224 with 41 subjects (control *n* = 14; 3.0 g/kg *n* = 14; 5.0 g/kg *n* = 13). Unlike the anxiety-like effect found in the first elevated plus maze session, CIEg during adolescence did not significantly produce anxiety-like behavior in male rats as measured by time away from the wall, i.e., not in thigmotaxis (Kruskal–Wallis test, KW = 1.972, *p* > 0.05). In addition, total movement was not altered by CIEg during adolescence (Kruskal–Wallis test, KW = 2.03, *p* > 0.05) (data not shown).

Finally, anxiety-like behavior was reassessed on PND 579 in the elevated plus maze (control *n* = 11; 3.0 g/kg ethanol *n* = 10; 5.0 g/kg ethanol *n* = 9). Unlike the initial assessment in the plus maze, a trend for less percent open arm entries (Kruskal–Wallis test, KW = 3.345, *p* > 0.05) and percent open arm time (Kruskal–Wallis test, KW = 3.046, *p* > 0.05) was found in animals administered ethanol during adolescence. However, no significant effects were found (all *p*’s greater than 0.05). To determine if aging itself may have impacted our ability to find an anxiety-like effect at PND 579, we analyzed plus maze behavior in the water treated control animals that were tested on the elevated plus maze on both PND 49 and PND 579. The normal aging process increased an anxiety-like behavioral phenotype whereas animals on PND 579 spent significantly less percent time on the open arms compare to the percent time spent on the open arms when they were PND 49 [paired *t*-test, *t* = 7.044, df(8), *p* < 0.01]. Therefore, a floor effect may have impacted our ability to find a significant anxiety-like effect produced by CIEg on PND 579.

#### Females

On PND 49, after accounting for subjects that either A. were not alive at this time point or B. fell off the maze, a total of 37 subjects (control, *n* = 12; 3.0 g/kg ethanol *n* = 12; 5.0 g/kg ethanol *n* = 13) were run on the plus maze. Unlike what was found in males, CIEg during adolescence did not significantly alter behavior on the elevated plus maze in females, in that no significant difference was found for percent open arm time (Kruskal–Wallis test, KW = 0.638, *p* > 0.05), percent open arm entries (Kruskal–Wallis test, KW = 4.168, *p* > 0.05), or total movement (Kruskal–Wallis test, KW = 0.891, *p* < 0.05).

Open field was determined on PND 244 with a total of 41 subjects (control *n* = 14; 3.0 g/kg *n* = 13; 5.0 g/kg *n* = 14). Similar to the initial elevated plus maze behavior in females, CIEg during adolescence did not significantly alter anxiety-like behavior as measured by time not in thigmotaxis (Kruskal–Wallis test, KW = 0.779, *p* > 0.05). In addition, total movement was not altered by CIEg during adolescence (Kruskal–Wallis test, KW = 0.932, *p* > 0.05) (data not shown).

Finally, anxiety-like behavior was reassessed on PND 579 in the elevated plus maze (control *n* = 12; 3.0 g/kg ethanol *n* = 11; 5.0 g/kg ethanol *n* = 12). Like that found on PND 49, CIEg during adolescence did not alter behavior on the elevated plus maze as measured by percent open arm time (Kruskal–Wallis test, KW = 0.1779, *p* > 0.05) and percent open arm entries (Kruskal–Wallis test, KW = 0.065, *p* > 0.05) (data not shown).

### Impact of chronic intermittent ethanol exposure during adolescence on spatial reference learning and behavioral flexibility late in life

#### Males

**FIGURE 2:**
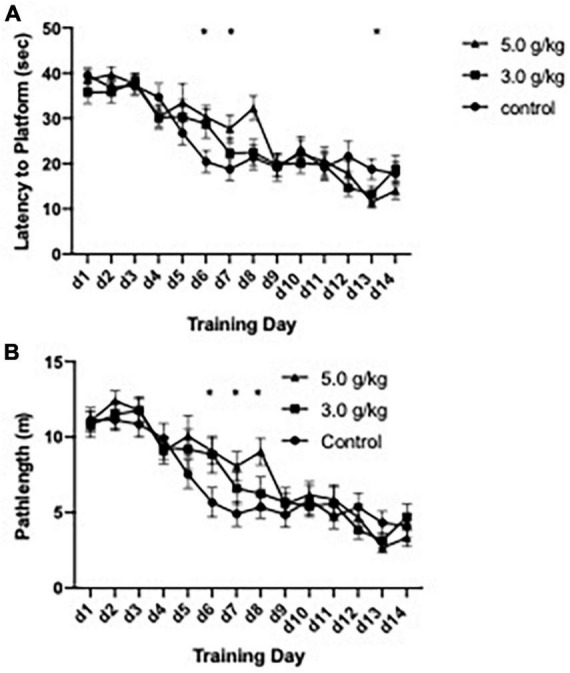
Chronic intermittent ethanol exposure via gavage during adolescence produces spatial learning deficits in male rats that are dose-dependent. Male rats were trained in the submerged version of the Morris water maze beginning on PND 579. Significant impairments due to ethanol exposure were found for latency to the platform **(A)** and for swim distance to the platform **(B)** in the 5.0 g/kg ethanol exposure group but not in the 3.0 g/kg ethanol exposure group. Data are mean performance with error bars denoting standard error of the mean. The symbol “*” denote *p* < 0.05, see text for exact value.

The impact of CIEg during adolescence on spatial reference learning was accessed via performance in the submerged platform version of the Morris water maze (Morris, 1981) beginning on PND 582, approximately 530 days following completion of the ethanol administration. One control animal was removed from the study due to minimal learning (average swim latency per day over 30 s on training days 1–10) resulting in a total of 34 animals being tested (control *n* = 11; 3.0 g/kg ethanol *n* = 12; 5.0 g/kg ethanol *n* = 11). CIEg during adolescence significantly altered spatial learning, as measured by swim latency to the submerged platform [two-way ANOVA with repeated measures, ethanol dose during CIEg (3) by training day (14), significant interaction of CIEg dose and training day, *F* = 1.64, df(26,403), *p* = 0.0261; significant main effect of day, *F* = 31.85, df(13,403), *p* < 0.0001]. The significant interaction was queried to determine the effect of CIEg during adolescence on spatial learning. Dunnett’s *post-hoc* tests revealed that 5.0 g/kg ethanol exposure during adolescence resulted in significantly worse performance on training day 6 (Dunnett’s *post-hoc* test, *q* = 3.98, *p* = 0.0197) and training day 8 (Dunnett’s *post-hoc* test, *q* = 3.98, *p* = 0.0198) compared to control treated animals and significantly better performance on training day 13 (Dunnett’s *post-hoc* test, *q* = 3.96, *p* = 0.0238) (see Figure 2).

Similar to the effects found with swim latency to the submerge platform, CIEg during adolescence produced a borderline significant impact on swim pathlength in the water maze [two-way ANOVA with repeated measures, ethanol dose (3) during CIE by training day (14), borderline significant interaction of CIEg dose and training day, *F* = 1.495, df(26,403), *p* = 0.058; significant main effect of day, *F* = 39.27, df(13,403), *p* < 0.0001]. *Post hoc* analysis supported the borderline effect demonstrating that CIEg during adolescence impaired spatial learning. Specifically, animals treated with 5.0 g/kg ethanol during adolescence had significantly longer swim pathlengths on training day 6 (Dunnett’s *post-hoc* test, *q* = 3.76, *p* = 0.0153), day 7 (Dunnett’s *post-hoc* test, *q* = 3.48, *p* = 0.0267) and day 8 (Dunnett’s *post-hoc* test, *q* = 4.07, *p* = 0.0082) while animals treated with 3.0 g/kg ethanol during adolescence had significantly longer swim pathlengths on training day 6 (Dunnett’s *post-hoc* test, *q* = 3.58, *p* = 0.0219).

**FIGURE 3:**
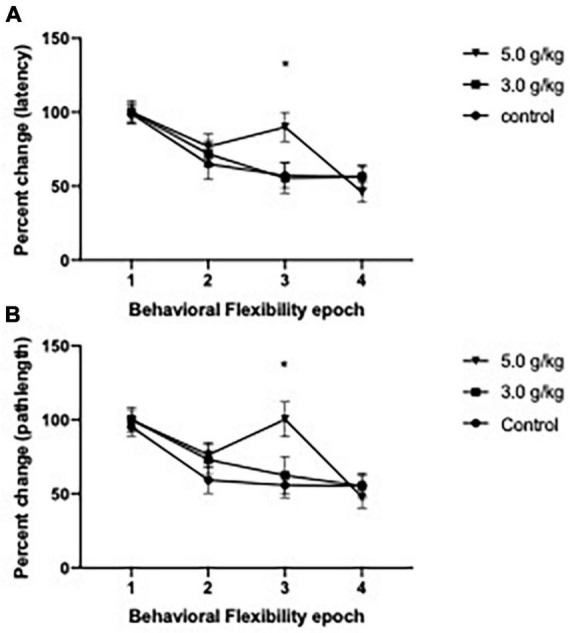
Chronic intermittent ethanol exposure via gavage during adolescence produces deficits in behavioral flexibility late in life in male rats. Male rats were trained for 2 days on a reversal platform task in the Morris water maze. Significant impairments due to ethanol exposure were found on performance for both percent change in swim latency **(A)** and for percent change in swim pathlength **(B)**. Data are mean performance with error bars denoting standard error of the mean. The symbol “*” denote *p* < 0.05, see text for exact value.

To investigate the effect of CIEg during adolescence on behavioral flexibility in aged animals, we utilized a reversal paradigm in the water maze for 2 days (a total of eight trials), whereas the platform was rotated 180° from its initial location. As previously done (Ho et al., 2022; Matthews et al., 2022), we treated the first two trials of reversal day 1 as learning trials and then analyzed percent change over the next six trials as a measure of behavioral flexibility, with these six trials grouped into two trial epochs. CIEg during adolescence impaired behavioral flexibility when animals were tested over 530 days later, as measured by latency to the submerged platform [two-way ANOVA with repeated measures, CIEg dose during adolescence (3) by reversal epoch (4), significant interaction of CIEg dose and epoch, *F* = 2.256, df(6,93), *p* = 0.0446]. *Post-hoc* tests confirmed that high dose CIEg during adolescence impaired behavioral flexibility in that animals treated with 5.0 g/kg ethanol during adolescence performed significantly worse on the third epoch of behavioral flexibility compared to the control animals (Dunnett’s *post-hoc* test, *q* = 2.503, *p* = 0.0388). In agreement with the latency data, CIEg during adolescence impaired behavioral flexibility, as determined by swim pathlength to the submerged platform [two-way ANOVA with repeated measures, CIEg ethanol dose during adolescence (3) by reversal epoch (4), significant interaction of dose and epoch, *F* = 2.399, df(6,93), *p* = 0.0336]. *Post-hoc* tests confirmed that high dose CIEg during adolescence impaired behavioral flexibility in that animals treated with 5.0 g/kg ethanol during adolescence performed significantly worse on the third epoch of behavioral flexibility compared to the control animals (Dunnett’s *post-hoc* test, *q* = 3.086, *p* = 0.019) (see Figure 3).

#### Females

Unlike the effect found in males, CIEg during adolescence did not significantly alter spatial reference learning in female rats when tested approximately 530 days following the completion of ethanol exposure, as determined by latency to the submerged platform [two-way ANOVA with repeated measures, CIEg dose during adolescence (3) by training day (14), main effect of day, *F* = 40.32, df(13,416), *p* < 0.0001] or swim pathlength to the submerged platform [two-way ANOVA with repeated measures, CIEg dose during adolescence (3) by training day (14), main effect of day, *F* = 59.69, df(13,416), *p* < 0.0001]. Furthermore, unlike what was found in males, behavioral flexibility was not significantly altered by CIEg exposure during adolescence in female rats, as determined by latency to the submerged platform [two-way ANOVA with repeated measures, CIEg dose during adolescence (3) by behavioral flexibility epoch (4), main effect of epoch, *F* = 15.69, df(3,128), *p* < 0.0001].

### [^3^H]PK11195 binding

**FIGURE 4:**
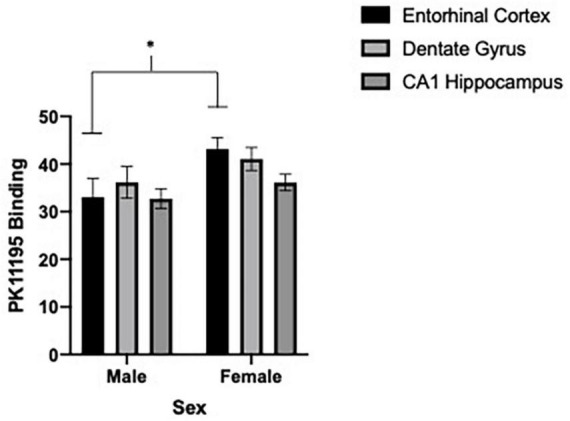
Sex-dependent effects of microglia activation late in life are brain region dependent with females having higher levels than males, specifically in the entorhinal cortex. Binding of PK11195 in three limbic brain regions in control treated female and male aged rats. Data are mean binding with error bars denoting standard error of the mean. The symbol “*” denote *p* < 0.05, see text for exact value.

**FIGURE 5:**
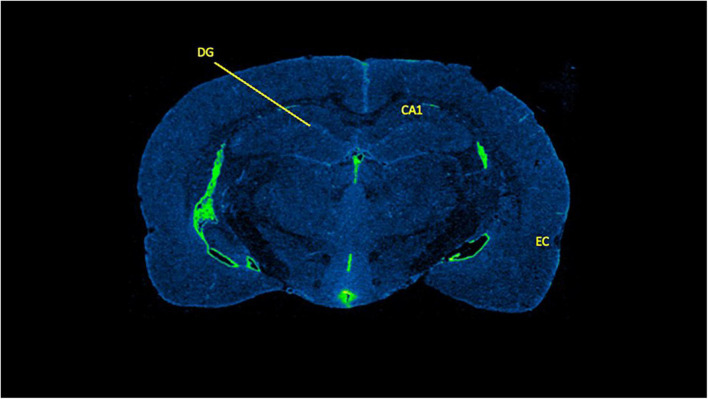
A representative data sample of [^3^H]PK11195 binding in brain. Brain slice is from a 5.0 g/kg ethanol treated female rat with the relevant brain regions labeled. DG, dentate gyrus; CA1, CA 1 region of the hippocampus; EC, entorhinal cortex.

We first sought to determine if differences in microglia reactivity at ∼20 months of age existed in the entorhinal cortex, dentate gyrus and CA1 region of the hippocampus by subjects’ sex. Due to shipping error, one 5.0 g/kg ethanol brain from females was not included in the analysis. To determine sex-dependent microglia reactivity, we analyzed the density of TSPO autoradiography from female and male brains in the water exposed condition. As previously reported, sex-dependent effects in microglia reactivity were found [two-way ANOVA, sex (2) by brain region (3), significant interaction of sex by brain regions, *F* = 4.10, df(2,16), *p* = 0.0364 and a significant main effect of brain region, *F* = 6.80, df(2,16), *p* < 0.0001]. We used Sidak’s multiple comparisons test to query the interaction and found a significant sex difference in TSPO expression in the entorhinal cortex [*t* = 2.6, df(24), *p* = 0.0464] (see Figures 4, 5). Due to the sex-dependent nature of microglia reactivity in aged animals, we analyzed females and males separately and sought to investigate if CIEg during adolescence impacts TSPO expression in these three brain regions. Interestingly, CIEg during adolescence did not alter TSPO density in the three brain regions of male rats [two-way ANOVA with repeated measures, CIEg (3) by brain region (3), significant main effect of brain region, *F* = 10.8, df(2,24), *p* = 0.0008] even though the dentate gyrus was found to have higher levels of TSPO expression than either the entorhinal cortex (Tukey’s *post-hoc* test, *q* = 3.733, *p* = 0.048) or the CA1 region of the hippocampus (Tukey’s *post-hoc* test, *q* = 7.799, *p* = 0.0002). For female rats, once again CIEg during adolescence did not alter TSPO density ∼20 months later in the three brain regions [two-way ANOVA with repeated measures, CIEg (3) by brain region (3), main effect of brain region, *F* = 40.27, df(2,22), *p* < 0.0001]. Furthermore, the pattern of TSPO expression by brain region was different in aged females compared to aged males in that the CA1 region of the hippocampus had significantly less TSPO expression compared to the entorhinal cortex (Tukey’s *post-hoc* test, *q* = 10.5, *p* < 0.0001) and the dentate gyrus (Tukey’s *post-hoc* test, *q* = 15.82, *p* < 0.0001) (see Table 1).

**TABLE 1:** Differential microglia reactivity by brain region in female and male rats late in life.

	Control	3.0 g/kg ethanol	5.0 g/kg ethanol
Female			
Entorhinal cortex	43.16 (2.4)	46.04 (2.0)	40.60 (3.5)
Dentate gyrus	41.06 (2.4)	44.16 (2.0)	42.40 (3.6)
CA1 hippocampus*	**36.12 (1.7)**	**37.50 (2.2)**	**35.30 (3.1)**
Males			
Entorhinal cortex	33.06 (3.9)	38.04 (3.7)	39.26 (2.5)
Dentate gyrus*	**36.16 (3.3)**	**41.44 (3.1)**	**40.34 (2.7)**
CA1 hippocampus	32.74 (2.0)	35.78 (1.9)	35.08 (1.6)

In female rats, less microglia reactivity was found in the CA1 region of the hippocampus compared to the entorhinal cortex or dentate gyrus while in male rats, greater microglia reactivity was found in the dentate gyrus compared to the CA1 region of the hippocampus or entorhinal cortex. Values are mean density and standard error of the mean are in the parenthesis. The symbol “*” denote *p* < 0.05, see text for exact value. The bolded values are significant from other groups by sex.

## Discussion

Adolescence represents a unique time period where significant brain maturation occurs (Bayer et al., 1982). In addition to changes in the nervous system, adolescence is a time when many individuals begin to experiment with new behaviors including alcohol consumption. Unfortunately, alcohol consumption in this age group is often high and occurs in a dangerous binge pattern (Patrick and Terry-McElrath, 2019). Animal research has been necessary to determine the impact of binge-like ethanol exposure in adolescent rats and has demonstrated significant negative effects, including increases in anxiety-like behaviors (Coleman et al., 2014; Van Skike et al., 2015; Varlinskaya et al., 2015; Vetreno et al., 2016; Kokare et al., 2017; Lee et al., 2017), impairments in cognition (Coleman et al., 2014; Gass et al., 2014; Barker et al., 2017; Fernandez et al., 2017; Contreras et al., 2019; Sey et al., 2019; Macht et al., 2020) and alterations in microglia reactivity (McClain et al., 2011, Grifasi et al., 2021; Melbourne et al., 2021; Silva-Gotay et al., 2021). In addition, previous research has shown that some effects of binge-like ethanol exposure during adolescence can last beyond the immediate time following exposure and into young adulthood (for review see Crews et al., 2019). However, little is known if these effects are transitory or if they last throughout most of the lifespan. Using both female and male rats, we provide evidence that chronic binge-like ethanol exposure during adolescence produces initial anxiety-like effects in male, but not female, rats. However, anxiety-like effects resolved as they were not found in adult or aged subjects of either sex. In addition, we provide data revealing a subtle, yet significant, impairment in reversal learning in aged male rats due to CIEg exposure during adolescence, an effect that remarkably was found several hundred days following the ethanol exposure during adolescence. Furthermore, CIEg during adolescence did not produce persistent microglia reactivity in either the entorhinal cortex, dentate gyrus or CA1 region of the hippocampus in male or female rats. Finally, we report that microglia reactivity was greater in female aged rats compared to male age rats in the entorhinal cortex but not the dentate gyrus or CA1 region of the hippocampus.

Chronic intermittent ethanol during adolescence has been shown to increase anxiety-like behaviors in rodents (Coleman et al., 2014; Van Skike et al., 2015; Varlinskaya et al., 2015; Vetreno et al., 2016; Kokare et al., 2017; Lee et al., 2017; Healey et al., 2021; See Crews et al., 2019 for review). Specifically, CIEg during adolescence in male rats produces a significant anxiety-like effect in the elevated plus maze that can last for several weeks (Pandey et al., 2015; Van Skike et al., 2015; Kokare et al., 2017; Kyzar et al., 2017), however increased in anxiety-like behavior is not always found (see Towner and Varlinskaya, 2020 for a review of this field). While it has been reported that CIE can produce a sex-dependent increase in social anxiety behavior (effect in males significantly greater than in females) when subjects are tested in young adulthood (Varlinskaya et al., 2015) few studies have investigated sex-dependent effects of CIE during adolescence. For example, CIEg during adolescence will produce differential behavioral effects on the elevated plus maze that are sex dependent (Healey et al., 2021). Specifically, ethanol exposure during adolescence results in increased open arm time in female rats, but not male rats at PND 70, suggesting altered anxiety-like effects due perhaps to a blunted or exaggerated response in females. Furthermore, late adolescence ethanol exposure also produces sex-specific effects whereas males, but not females, have an anxiety-like phenotype while early adolescence ethanol exposure produces an anxiety-like effect in both sexes (Towner and Varlinskaya, 2020). However, the sex-specific effect in anxiety-like behavior due to adolescent ethanol exposure has not always been reported (Amodeo et al., 2018; Gamble and Diaz, 2020). The current work helps clarify some of these issues while also producing additional questions that require further research. First, the results from the current study support previous research that demonstrated CIE throughout the majority of adolescence can result in sex-specific effects where male subjects display an anxiety-like effect while females do not display an anxiety-like effect. Second, the results provide important information concerning the temporal limitations of any anxiety-like effect produced by CIEg during adolescence. Specifically, we did not find a significant anxiety-like effect in the open field or elevated plus maze on PND 244 or PND 579. Third, any resultant residual anxiety-like effect that does exist late in life will be very subtle, requiring a large number of animals to have sufficient statistical power. Finally, the natural increase in anxiety-like behavior over the course of normal aging (Imhof et al., 1993) and present results with males) will make teasing apart any long lasting anxiety-like effect late in life by CIEg during adolescence difficult.

The impact of CIE on cognition late in life has not been extensively investigated. While previous work has shown that CIE can impair cognition in young adulthood (for review see Crews et al., 2019) or into middle age (Reitz et al., 2021), we are aware of only two studies that investigated the effect of CIE during adolescence in aged rats (Matthews et al., 2017). In the first study, male rats were exposed to ethanol or to saline during adolescence and then tested over a 500+ day longitudinal study. CIE during adolescence coupled with an ethanol challenge at certain time points during the lifespan resulted in delayed spatial learning, as demonstrated by swim latency to the platform. However, the effect of CIE in female subjects was not determined nor was the impact of CIE on other cognitive measures, such as behavioral flexibility (Matthews et al., 2017). In a more recent project (Matthews et al., 2022), CIEg during adolescence in both female and male subjects resulted in altered behavioral flexibility, but the pattern of the impairment was sex dependent. Specifically female subjects administered 5.0 g/kg ethanol had impaired behavioral flexibility throughout their life (a main effect of ethanol exposure during adolescence) while males administered 5.0 g/kg ethanol primarily had impaired behavioral flexibility only later in life (a significant interaction of ethanol dose and aging).

In the current project, CIEg during adolescence in males produced alterations in spatial learning and impairments in behavioral flexibility late in life, but did not alter spatial learning nor behavioral flexibility in females late in life. Previous work has demonstrated that CIE during adolescence can produce impairments in the swimming version of the radial arm maze task (tested at ∼115 PND) and novel object recognition (tested at ∼210 PND) in both male and female rats (Galaj et al., 2019) and in male rats during middle age (Reitz et al., 2021). The current work supports the recently reported impairment in behavioral flexibility found in male rats (Matthews et al., 2022). However, the lack of an impairment in behavioral flexibility in the female subjects in the current work was surprising. Several possibilities exist to explain the differential results. First examination of Figure 5C and D from Matthews et al. (2022) shows that female subjects had similar performance on the behavioral flexibility test late in life regardless of the ethanol dose (or lack thereof in the control subjects). This suggests that CIEg during adolescence in female subjects produces impairments in female subjects early in life but the normal aging process occludes the effect late in life. Second, animals in the current project were only tested in the water maze once late in life instead of multiple times throughout life as previously done (Matthews et al., 2022). Future research is needed to better understand the impact of CIEg during adolescence on cognitive performance in female subjects. In addition, research is needed to understand the effect of CIEg during adolescence on episodic type tasks in the water maze [i.e., spatial working memory tasks (Foster, 2023)].

Alcohol-induced increases in microglia reactivity as determined by [^3^H]PK11195 autoradiography for TSPO expression, was not detectable 20-months following CIEg administration in either male or female subjects. Alcohol exposure produces microglia reactions in adolescents (McClain et al., 2011; Vetreno and Crews, 2012; Barton et al., 2017; see also Melbourne et al., 2021 for review), but assessment of TSPO has only been reported in abstract form (Van Doorn et al., in preparation). However, we did find increased microglia reactivity in female rats compared to male rats although the effect was selective to brain region in that [^3^H]PK11195 binding was greater in the entorhinal cortex compared to either the dentate gyrus or CA1 region of the hippocampus. This finding may reflect that females have greater amounts of microglia compared to males in some limbic regions including greater amounts in the dentate gyrus and CA1 region of the hippocampus of mice though female rats tend to have fewer microglia than males, an effect which does vary across limbic regions and with aging (Perkins et al., 2018; Ince et al., submitted). A heightened state of reactivity has been found in females, but there is significant variability in the literature based on what was measured (Bollinger et al., 2015; reviewed in Cortez et al., 2020; Lynch, 2022; Ugidos et al., 2022). While microglia have greater reactions to alcohol in adult female Sprague Dawley rats (Barton et al., 2017) and in F344 adult rats that consume ethanol for several months, the opposite was true for Wistar strain rats where Toll-like receptor-4 gene expression was higher in the males after alcohol drinking (Silva-Gotay et al., 2021). Intriguingly, sex differences in neuroimmune signatures are found at the level of the brain transcriptome, even when animals are phenotypically similar for high alcohol drinking (Hitzemann et al., 2022). In aging, the opposite may be true: males appear to have amplified inflammatory priming of microglia which may underlie their age-associated cognitive deficits (Ince et al., submitted). Further, CIE during adolescence can produce sex-specific effects in adulthood on hippocampal glial cells suggesting adolescence is a vulnerable period when ethanol exposure can alter glial expression and function. Thus, future research should investigate if differential microglia reactivity or priming exists between CIEg treated adolescent male and female animals and CIEg treated aged male and female animals.

Currently, it is unknown what are potential neurobiological mechanisms producing impaired cognition late in life following CIEg during adolescence. However, studies have provided potential brain mechanisms that are altered by exposure during adolescence and last into adulthood. For example, exposure to ethanol during adolescence produces a significant reduction in cholinergic interneurons in the nucleus accumbens of male and female rats (Galaj et al., 2019) and altered cholinergic activity in the orbital frontal cortex (Kipp et al., 2021). Further, CIE during adolescence can reduce efflux of acetylcholine 12–14 months later and the reduction is selective to male rats (Reitz et al., 2021). Given it has long been proposed that reductions in cholinergic function may underlie a portion of the cognitive decline observed in aging (Schliebs and Arendt, 2011 for review) it is interesting to speculate that CIEg during adolescence may result in increased cholinergic disfunction thereby leading to larger cognitive deficits late in life. Future studies are needed to test this hypothesis.

Previously, we have reported that CIEg during adolescence in male rats but not female rats results in a significant decrease in survivability over a ∼22 month experimental period compared to control animals (Matthews et al., 2022). In the current project, we did not find a significant effect in survivability based on sex or ethanol exposure during adolescence. However, the point in time at which male subjects in our previous work (Matthews et al., 2022) began to be removed from the study due to either A. death or B. a significant health concern began at approximately 21 months of age. Future studies should systematically investigate the time point between 19 and 22 months of life following CIEg during adolescence. This time period may represent a critical window that results in differential sex-specific health issues in subjects exposed to ethanol during adolescence.

The current results demonstrate not only sex and age-dependent effects but also a significant dose effect, particularly in male subjects. For example, 5.0 g/kg ethanol, but not 3.0 g/kg ethanol, produced an initial anxiety-like effect in male rats. In a similar vein, 5.0 g/kg ethanol altered initial spatial learning and impaired behavioral flexibility only in male rats while the 3.0 g/kg ethanol dose did not alter cognition. These data suggest that male rats may be more sensitive than female rats to the long-term cognitive impairing effects of ethanol. However, the current work has several limitations. First, the studies are underpowered statistically. Specifically, it was found that 5.0 g/kg CIEg in male rats produced a very strong trend toward an impairment in spatial learning based on swim pathlength to the platform in that the significance value for the overall ANOVA was a 0.058. This likely was due to reduced animal numbers because of removal of some animals due to health reasons or death. Given the exploratory *post-hoc* tests all confirm that the 5.0 g/kg ethanol exposed animals performed worse than control animals and mirrors the significant effects found with swim latency to the platform, it is likely a valid and reliable spatial learning impairment. Second, anxiety-like behavior in animals increases with age (Imhof et al., 1993; current results in male rats) resulting in a floor effect for common dependent variables in the elevated plus maze such as percent open arm time or open arm entries. This floor effect makes it more difficult to determine if CIEg during adolescence results in an increased anxiety-like effect in aged animals. It is likely the development of new experimental designs and/or apparatus will be needed to determine the impact of CIEg during adolescence on anxiety-like behavior in aged animals. Third, few people drink in a binge-pattern during adolescence and then do not consume ethanol again throughout their lifespan. As such the research strategy employed here does not model the typical drinking history of older adults. We employed this strategy to capitalize on the extensive research already conducted that investigates the impact of adolescent ethanol exposure during adolescence and then testing in adulthood (see Crews et al., 2019 for a review of this work). The current work greatly extends these findings into the aging portion of the lifespan. However, it is critical to model alcohol use across the lifespan. These studies are being initiated in our laboratory.

## Conclusion

In conclusion, CIEg during adolescence produced anxiety-like behavior in male rats, but not female rats, on the elevated plus maze the day following completion of the ethanol exposure regime. However, the anxiety-like behavioral effect was not seen on PND 224 or PND 579 suggesting the effects are transitory as it relates to the lifespan. Further, when tested on PND 582, male rats, but not female rats, administered 5.0 g/kg ethanol CIEg during adolescence had subtle, yet significant, impairments in spatial learning and behavioral flexibility. Finally, CIEg during adolescence did not alter microglia expression in aging. Further work is needed to determine what brain mechanisms underlie these effects and what is the impact of ethanol exposure across the lifespan.

## Data availability statement

The raw data supporting the conclusions of this article will be made available by the authors, without undue reservation.

## Ethics statement

The animal study was reviewed and approved by the University of Wisconsin–Eau Claire IACUC.

## Author contributions

SS, ST, AS, GR, BI, QP, and AK collected and edited the data. DM designed the study, analyzed the data, and wrote the manuscript. JP collected and edited the data. KN designed the study, and wrote and edited the manuscript. All authors contributed to the article and approved the submitted version.
